# CoreGenes5.0: An Updated User-Friendly Webserver for the Determination of Core Genes from Sets of Viral and Bacterial Genomes

**DOI:** 10.3390/v14112534

**Published:** 2022-11-16

**Authors:** Patrick Davis, Donald Seto, Padmanabhan Mahadevan

**Affiliations:** 1 Department of Biology, The University of Tampa, Tampa, FL 33606, USA; 2 Department of Systems Biology, George Mason University, Manassas, VA 20110, USA

**Keywords:** coregenes, webserver, bioinformatics, genomics, viruses, bacteria

## Abstract

The determination of core genes in viral and bacterial genomes is crucial for a better understanding of their relatedness and for their classification. CoreGenes5.0 is an updated user-friendly web-based software tool for the identification of core genes in and data mining of viral and bacterial genomes. This tool has been useful in the resolution of several issues arising in the taxonomic analysis of bacteriophages and has incorporated many suggestions from researchers in that community. The webserver displays result in a format that is easy to understand and allows for automated batch processing, without the need for any user-installed bioinformatics software. CoreGenes5.0 uses group protein clustering of genomes with one of three algorithm options to output a table of core genes from the input genomes. Previously annotated “unknown genes” may be identified with homologues in the output. The updated version of CoreGenes is able to handle more genomes, is faster, and is more robust, providing easier analysis of custom or proprietary datasets. CoreGenes5.0 is accessible at coregenes.org, migrating from a previous site.

## 1. Introduction

Core genes are the set of common genes in a set of genomes, in contrast to genes which are not common amongst these genomes (accessory genes) [1]. A better understanding of these core genes has led to the design and synthesis of a minimal bacterial genome [2] to address basic and applied (biotechnological) questions and needs. Core genes have also revealed insights into carbon cycling and carbohydrate metabolism in soil metagenomes [3]. The variation in core genes can also be used for epidemiological typing in bacteria [4]. Core genes have also been used in evolutionary studies of nucleo-cytoplasmic large DNA viruses of eukaryotes [5].

With the growing bacterial and viral genome databases, there has been an increase in the demand for easily accessible and user-friendly software for genomic analyses. Since its original development in 2002 [6], CoreGenes has been continuously updated in order to increase its ease of use, add functionality, and meet additional user suggestions [7]. In particular, it has been used extensively for characterizing and classifying bacteriophages, for example resolving taxonomic issues in the *Podoviridae*, *Myoviridae*, and *Siphoviridae* families [8,9,10] and characterizing newly sequenced bacteriophages [11]. The taxonomic approach pioneered by the CoreGenes application for the *Podoviridae*, *Myoviridae*, and *Siphoviridae* families has been extended by other researchers [12]. CoreGenes has demonstrated utility in the data mining of pathogenic viral and bacterial genomes [13]. However, a limiting factor with older versions of the software was its slower processing speed, which required a smaller allowance for accession numbers in each run and greatly limited the size of the input genomes. In this iteration of CoreGenes, these limitations have been greatly improved. Additionally, coding sequence retrieval can now be used on the web interface, allowing the user to easily view the genome or retrieve new coding sequence information quickly, bypassing the NCBI webpage. 

## 2. Methods

CoreGenes5.0 is written mainly using Python for processing. The webpage implementation is written using Python’s Django module, HTML, CSS, and JavaScript. The reimplementation of the CoreGenes3.5 algorithm uses the same iterative algorithm to process a query genome against a reference genome, and to then create a new consensus genome as described previously [7]. The updated 3.5 version uses MMseqs2 [14] for rapid protein searches instead of Washington University BLAST (WU-BLAST). The genomes are retrieved from GenBank using the user-inputted accession numbers. The former option for the user-input blast score has been updated to accept e-values. While the former iteration of CoreGenes only allowed for up to five input accession numbers, both versions of the updated CoreGenes algorithm now allow for the quick input of twenty accession numbers or an uploaded .txt file of up to two hundred comma-separated accession numbers, using the file upload option. It is recommended that protein queries not exceed 50 input genomes for bacterial genomes, due to their larger size requiring longer processing.

CoreGenes5.0 uses the GET_HOMOLOGUES package [15,16] with BLAST+ [17,18] to perform group protein clustering using default options. The group protein clustering is supported by three common clustering algorithms: OrthoMCL [19], bidirectional best hit, and COGtriangles [20]. In this updated version, an optional input for the inclusion or exclusion of paralogs is available. This option excludes sequences that have significant matches in the same genome and is implemented by the GET_HOMOLOGUES package. The GET_HOMOLOGUES manual defines “inparalogs” as “sequences with best hits in its own genome and excludes clusters with these sequences”. In addition, the user can also input a BLAST e-value in the web interface. The output table of either version is displayed on the webserver, with a link that is available for up to one month. A .csv file with a similar table formatting is available for download through the optional email input. For queries which may take longer to process, an optional email notification is now available with a link to the results page. 

CoreGenes now includes a coding sequence (CDS) retrieval option. Genome accession numbers are input, then used to parse coding sequence data from the Genbank database. The parsed CDS files can be downloaded in a zipped folder containing the FASTA files (with a .fasta extension) of the input genomes via email. The Custom Dataset upload option has been updated to accept a standardized FASTA file, with a space-separated accession identifier and protein title. 

The rewritten Iterative Comparison Algorithm with MMseqs2 allows for a higher number of accession numbers to be processed. While the iterative comparison algorithm is able to handle larger genomes than the previous version of CoreGenes, the algorithm still performs with greater speed when using viral and small bacterial genomes. The display table is formatted in an easy-to-read format, with hyperlinks for each accession number to the NCBI page. Hypothetical proteins are highlighted in red for an easy-to-locate comparison with annotated homologues. For larger bacterial genomes, the CoreGenes5.0 webserver is able to process multiple 5 Mb genomes in minutes and is able to handle genomes as large as 10 Mb. The main differences between CoreGenes5.0 and the previous version, CoreGenes4.0, are shown in Table 1 below.

## 3. Results

**Figure 1:**
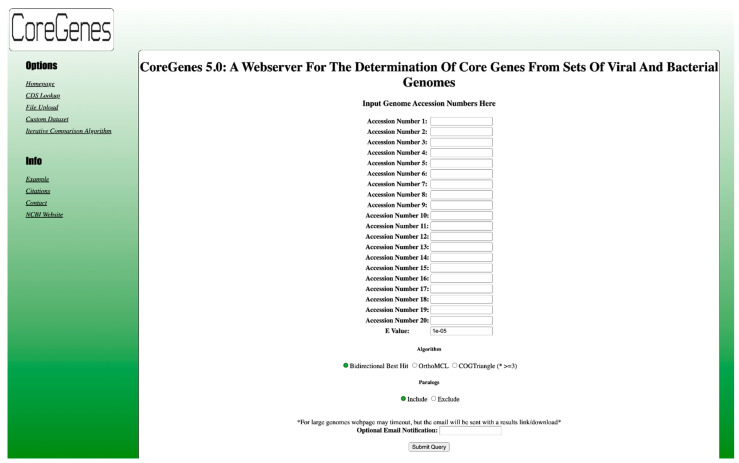
Web interface for CoreGenes5.0. Up to 20 accession numbers can be entered, as well as an optional email address for results. The bidirectional best hit, OrthoMCL, or COGTriangle algorithms can be chosen. The original CoreGenes3.5 iterative comparison algorithm can be chosen by clicking on the link on the left, as this has been used extensively in bacteriophage taxonomic analyses. Lists of accession numbers and custom datasets can also be uploaded by clicking on the links on the left.

**Figure 2:**
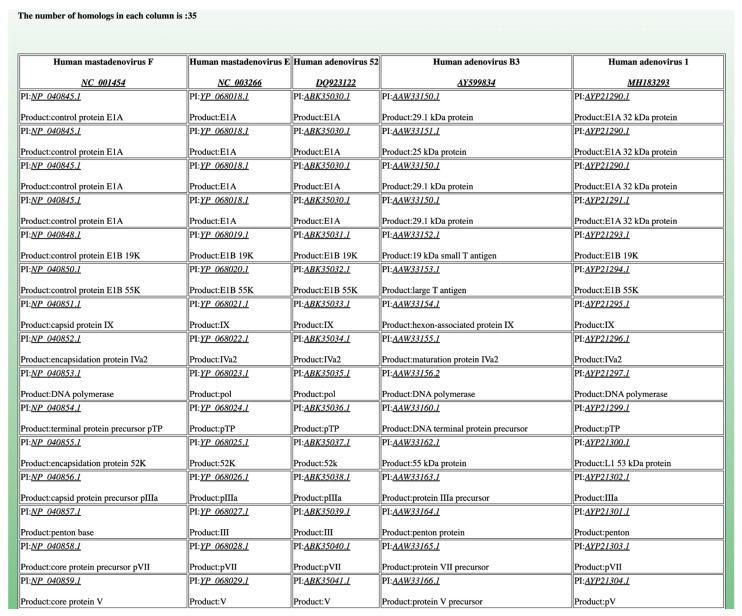
Partial CoreGenes output from five human adenovirus genomes. The total number of core genes found is 35. Links to the complete genomes and individual proteins are also provided for additional analysis.

The web interface of CoreGenes5.0 (Figure 1) enables the input of 20 genome accession numbers. We have designed the user interface to be intuitive and easy to use, without a lot of confusing options. Links on the left of the page lead to the file upload of accession numbers for batch processing of more than 20 genomes. Custom datasets can also be uploaded using the link on the left. The “old” CoreGenes3.5 algorithm can also be accessed by a link on the left (Iterative Comparison Algorithm). Figure 2 shows the partial CoreGenes5.0 output of five human adenovirus genomes. The output is clean and easy to read for the human eye. Links can be clicked on to access the complete genome or individual proteins in GenBank.

**Figure 3:**
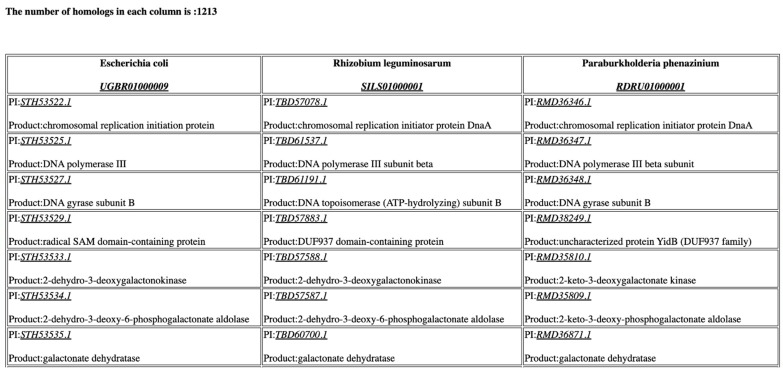
Partial CoreGenes output from three bacterial genomes approximately 5 Mb in size. The links to the complete genomes and individual proteins are shown. As noted, the number of core genes identified is 1213.

CoreGenes5.0 can process small or large bacterial genomes in minutes, as shown in Table 2. Three 5 Mb bacterial genomes take less than 10 minutes to process, while three 1 Mb genomes take only a minute to process. It must be noted that these times will increase as the number of queried genomes increase. A partial core gene output of three 5 Mb bacterial genomes is shown in Figure 3.

**Figure 4:**
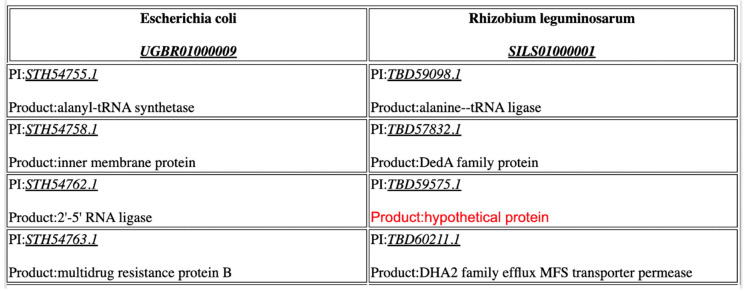
CoreGenes assists in the identification and annotation of hypothetical proteins. Here, the annotated hypothetical protein in *Rhizobium leguminosarum*, labeled in red, is putatively identified as 2’-5’ RNA ligase in a reference genome, *E. coli*. Flagging this to the user allows for a subsequent evaluation for identity.

The annotation of hypothetical or previously annotated “unknown” proteins is made possible by highlighting all hypothetical proteins in red and by providing putative homologues across the output table. For example, in Figure 4, a hypothetical protein in the bacterium *Rhizobium leguminosarum* is annotated as a hypothetical protein and labeled in red in the right-hand column. The homologous protein in *E. coli*, which is annotated as a 2’-5’ RNA ligase (e-value threshold ≤1 × 10^−5^), is located on the same row in the left-hand column. It is very likely that this hypothetical protein is also a 2’-5’ RNA ligase. Theoretically, a genome with hundreds of hypothetical proteins may be annotated using a closely related reference genome and CoreGenes5.0.

## 4. Discussion

CoreGenes5.0 is a vastly improved version of its predecessor, CoreGenes3.5 [7], which is no longer supported at its previous home (http://binf.gmu.edu/genometools.html, accessed on 9 November 2022). It is strongly recommended that users migrate to this updated version. This version is more user-friendly, faster, more robust, and able to handle more genomes, all of which were suggested by users. The bacteriophage research community has used CoreGenes extensively to resolve taxonomic issues that were in question, based on traditional methods. Nucleotide sequence analysis for taxonomy has been improved with the application of this tool [21]. CoreGenes has been cited in 334 publications, as per Google Scholar. Frequent citations are in the International Committee on Taxonomy of Viruses (ICTV) Working Group publications, which are not recorded by Pubmed (https://pubmed.ncbi.nlm.nih.gov, accessed on 9 November 2022). For example, Kropinski A.M., Turner D., Tolstoy I., Moraru C., Adriaenssens E.M., and Mahony J. cited results from CoreGenes 3.5 in “Code assigned: 2022.001 B” as a report from the Bacterial Viruses Subcommittee, Caudoviricetes Study Group in 2022. This publication notes that “the genera Sfi21dtunalikevirus (2013.036 a-dB) and Sfi1unalikevirus (2013.034 a-dB) were renamed Moineauvirus and Brussowvirus, respectively through Taxonomy Proposal 2015.025 aB”. These examples serve to underscore the usefulness and current application of CoreGenes.

CoreGenes5.0 provides large-sized bacterial genomes analyses in a shorter timeframe as well. In addition, results can be downloaded in .csv format for offline use. Batch processing is available by uploading a list of accession numbers, with the results emailed to the user. Hypothetical proteins can also be readily identified and annotated, using reference genomes. An added option of including/excluding paralogs may be of particular interest to CoreGenes5.0 users. This was conveniently a function of the GET_HOMOLOGUES package [15,16]. Its definition of “paralogs” is used, as noted in Methods. All of these features make CoreGenes5.0 an easy-to-use software tool for non-computationally savvy users.

Future work will involve replacing the BLAST+ portions of the pipeline with MMseqs2 [14] or DIAMOND [22] to perform fast protein searches. This will enable even more genomes to be processed at a faster rate. Eventually, we hope to transfer the application to a more powerful computer server or a cloud computing environment for higher throughput processing. It should be noted that CoreGenes has been in continuous use since the earliest version in 2002 [6,23].

## Figures and Tables

**Table 1:** Additional functionality provided by CoreGenes5.0 compared to CoreGenes4.0.

Functionality	CoreGenes4.0	CoreGenes5.0
Additional clustering algorithms such as Bidirectional best hit, OrthoMCL and COGtriangles made available through the GET_HOMOLOGUES package	X	✓
Faster protein searches using MMseqs2 in the Iterative Comparison Algorithm	X	✓
Email results to user	X	✓
Easy CDS retrieval from GenBank	X	✓
More robust custom data input	X	✓

**Table 2:** CoreGenes5.0 analysis of three bacterial genomes. Five runs were completed, with different but uniform genome sizes for each run. The data were processed using the Bidirectional Best Hit method, paralog inclusion, and an e-value of 1 × 10^−5^.

Genome Size	1 Mb	2 Mb	3 Mb	4 Mb	5 Mb
**Accession #s**	NUHQ01000006.1 CAIT01000004.1 ASWA01000004.1	MTBP01000002.1 CP033822.1 UHGI01000001.1	UGNN01000001.1 CP035563.1 UKAD01000001.1	CP021892.1 CP012872.1 UGNN01000001.1	UGBR01000009.1 SILS01000001.1 RDRU01000001.1
**Run Time**	00 h:01 m:10 s	00 h:02 m:22 s	00 h:05 m:00 s	00 h:05 m:54 s	00 h:09 m:56 s
**Number of Homologues**	41	372	499	732	1213

## Data Availability

The genome data analyzed in this paper are available from GenBank at ncbi.nlm.nih.gov.

## Availability and Requirements

Server’s homepage: coregenes.org. Operating system(s): Platform-independent. Other requirements: A web browser such as Chrome, Firefox, Safari, or Microsoft Edge is needed to access the webserver.
